# 40 years of progress in female cancer death risk: a Bayesian spatio-temporal mapping analysis in Switzerland

**DOI:** 10.1186/s12885-015-1660-8

**Published:** 2015-10-09

**Authors:** Christian Herrmann, Silvia Ess, Beat Thürlimann, Nicole Probst-Hensch, Penelope Vounatsou

**Affiliations:** 1 Cancer Registry St. Gallen-Appenzell, St Gallen, Switzerland; 2 Department Epidemiology and Public Health, Swiss Tropical and Public Health Institute, Basel, Switzerland; 3 University of Basel, Basel, Switzerland; 4 Department of Medical Oncology-Haematology, Kantonsspital St. Gallen, St. Gallen, Switzerland; 5 Breast Centre, Kantonsspital St. Gallen, St. Gallen, Switzerland

**Keywords:** Neoplasm, Breast cancer, Ovarian cancer, Cervical cancer, Uterine cancer, Switzerland, Bayesian inference, Disease mapping, Time trends

## Abstract

**Background:**

In the past decades, mortality of female gender related cancers declined in Switzerland and other developed countries. Differences in the decrease and in spatial patterns within Switzerland have been reported according to urbanisation and language region, and remain controversial. We aimed to investigate geographical and temporal trends of breast, ovarian, cervical and uterine cancer mortality, assess whether differential trends exist and to provide updated results until 2011.

**Methods:**

Breast, ovarian, cervical and uterine cancer mortality and population data for Switzerland in the period 1969–2011 was retrieved from the Swiss Federal Statistical office (FSO). Cases were grouped into <55 year olds, 55–74 year olds and 75+ year olds. The geographical unit of analysis was the municipality.

To explore age- specific spatio-temporal patterns we fitted Bayesian hierarchical spatio-temporal models on subgroup-specific death rates indirectly standardized by national references. We used linguistic region and degree of urbanisation as covariates.

**Results:**

Female cancer mortality continuously decreased in terms of rates in all age groups and cancer sites except for ovarian cancer in 75+ year olds, especially since 1990 onwards.

Contrary to other reports, we found no systematic difference between language regions. Urbanisation as a proxy for access to and quality of medical services, education and health consciousness seemed to have no influence on cancer mortality with the exception of uterine and ovarian cancer in specific age groups. We observed no obvious spatial pattern of mortality common for all cancer sites.

Rate reduction in cervical cancer was even stronger than for other cancer sites.

**Conclusions:**

Female gender related cancer mortality is continuously decreasing in Switzerland since 1990. Geographical differences are small, present on a regional or canton-overspanning level, and different for each cancer site and age group. No general significant association with cantonal or language region borders could be observed.

**Electronic supplementary material:**

The online version of this article (doi:10.1186/s12885-015-1660-8) contains supplementary material, which is available to authorized users.

## Background

Female gender related cancers, in particular cancer of the breast, corpus uteri, ovary and cervix uteri account for more than 40 % of newly diagnosed cancers and for about 30 % of cancer related deaths in Swiss women [1]. In the past decades, female cancer mortality declined in Switzerland and the more developed countries [1] mainly due to advances in the understanding of tumour biology and in early detection, as well as the introduction of targeted therapies. However, differences in the decrease within Switzerland have been reported, such as for breast cancer in four selected cantons [2].

Switzerland is a small, affluent and culturally diverse confederation of 26 relatively autonomous states called cantons. Health care policies are developed at cantonal level resulting in a large geographical variation in health expenditures, control programs and care planning. I.e. population based mammography screening programs were and are implemented at very different time points over a period of more than 20 years in the various cantons. Most studies, including the above, investigated differences on the same regional level –cantons–, but it remained unknown whether these are consistent geographical disparities related to cantonal decisions or artefacts due to the choice of geographical and time units; driven by sub regions or complete region. The only more detailed maps of female cancer mortality rates are those of Schüler and Bopp [3] depicting geographical variation in mortality during 1970–1990 on the basis of so called MS-regions, 106 ‘unofficial’ regions smaller than cantons defined by mobility considerations. Since they have not applied temporal and geographical smoothing, the results may be distorted especially in areas where the population is small. This makes it difficult to distinguish chance variability from real differences. To our knowledge, covariate-adjusted and smooth, nationwide maps of female cancer mortality depicting the changes over time and space are not available.

Therefore, we studied geographical and temporal trends of breast, ovarian, cervical and uterine cancer mortality in Switzerland, adding 20 years of data to previous work, using state-of-the-art methodology for results with more detail and fewer artefacts, and without prejudice of geographical unit or shape of time trends. Hence, we used the most detailed available data (municipality level) and accounted for non-linear time trends. We hypothesized similar patterns for the different cancer sites and/or age group. Bayesian spatial models are the state-of-the-art modelling approach for assessing spatio-temporal patterns and trends. They “smooth” or improve estimation of an unstable rate by “borrowing” strength from its neighbours [4]. They can also assess the significance of risk factors taking into account the geographical correlation, and are able to show spatial patterns after adjustment for geographical differences in certain risk factors.

## Methods

### Data sources

Female cancer mortality data was obtained for the period 1969–2011 from death certificates coded centrally by the Swiss Federal Statistical office (FSO). The data include age at death, year of birth and death for each individual, nationality, municipality of residence, the cause of death and co-morbidities. Cause of death and co-morbidities are coded using the 8th revision of the International Classification of Diseases (ICD) until 1994/1995 and afterwards using the 10th revision. The transition to the 10th revision of the ICD-10 was accompanied by changes in death certificate coding practices (priority rules). We used age- and cancer site-specific correction factors as proposed by Lutz et al. [5] for the death counts. We included all cases coded with main causes of death being cancer of the female breast (ICD-10 C50.0-C50.9), cervix (ICD-10 C53.0- C53.9), corpus uterine (ICD-10 C54.0-C55.9) and ovary (ICD-10 C56.9). According to federal regulations, mortality data excluding any identifiable information can be used in epidemiological studies without additional ethics committee approval.

Detailed population data on municipality level is only available from census that takes place in Switzerland every 10 years with the last one taking place in 2010. We aggregated the mortality data in five 4-year periods around the census years, i.e. 1969–1972, 1979–1982, 1989–1992, 1999–2002 and 2008–2011, in which population was assumed to be constant.

**Fig. 1:**
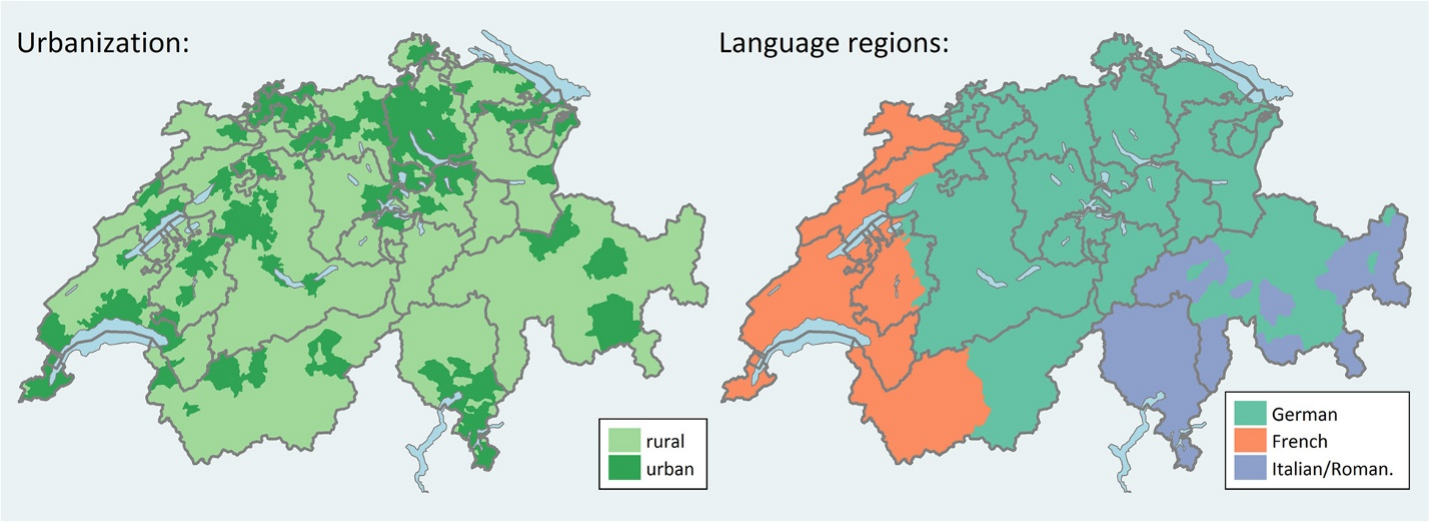
Urbanization classification and language regions in Switzerland

There are around 2,500 municipalities in the country. Over the study period, the number of municipalities has changed due to fusion, separation, deletion or new occurrences. We aligned all data on the 2011 municipality structure using spatial data for 2011 and municipality transition protocols for each year obtained from the FSO. From the same source, we retrieved data on language region (German, French and Italian/Romansh) and urbanisation (Fig. 1). We grouped municipalities classified as central agglomeration city, greater agglomeration and isolated city into “urban” leaving the classification “rural” unchanged.

### Statistical methods

Age was grouped into three groups (<55, 55–74, 75+ year olds). The geographical unit of analysis was the municipality.

In a preliminary analysis, we investigated SMR ratio values in a non-spatial model. Spatio-temporal Poisson and negative binomial regression models were fitted separately for each age group on the number of deaths aggregated by municipality and year with the mean being equal to the product of the expected death count and age standardised mortality rate. Indirect standardisation used 5 years age intervals. Expected mortality counts for each municipality, year and age group were obtained from the study population using nationwide age-specific mortality rates for all periods.

Space and temporal random effects as well as possible non-linear temporal trends were modelled on the log of the mean standardised mortality rate following model formulations of Jürgens et al. [6] (cf. Appendix 1). In particular, municipality-specific random effects were modelled via conditional autoregressive (CAR) models to filter out the noise and highlight the observed patterns. The models were formulated as hierarchical Bayesian models with parameter estimation via Markov chain Monte Carlo simulation (MCMC). We used the Deviance Information Criterion (DIC) to select the regression models from Poisson/Negative binomial regression with or without an additional set of unstructured random effects for each municipality.

Data on language and urbanisation were included as covariates in the model. These analyses will indicate whether there are statistically significant differences in the cancer mortality for each one of the above covariates, assessed by 95 % Bayesian Credible Intervals (CI).

From the estimates of the model, we produced smoothed maps displaying geographical patterns of female gender cancer mortality for each age group, cancer site and year since 1969 till recent almost to date.

## Results

Table 1 shows the number of female cancer deaths and crude rates per 100,000 person years in Switzerland by age group within the 4-year periods under investigation. Among the cancer sites studied, breast cancer was the most common cause of death, followed by ovarian, uterine and cervical cancer.

**Table 1:** Female cancer mortality in Switzerland by age group and time period corrected for coding changes

	Aged <55		Aged 55-74		Aged 75+	
Period	Total number of cases	Rate per 100,000 PY	Total number of cases	Rate per 100,000 PY	Total number of cases	Rate per 100,000 PY
Breast cancer						
1969-1972	995	10.3	2,185	89.5	997	161.6
1979-1982	1,062	11.1	2,336	92.6	1,556	169.5
1989-1992	1,110	11.0	2,345	89.0	2,512	210.6
1999-2002	908	8.6	2,184	74.9	2,169	159.4
2007-2010	813	7.4	2,303	68.1	2,501	160.9
Cervical cancer						
1969-1972	324	3.3	465	19.1	186	30.1
1979-1982	227	2.4	389	15.4	212	23.1
1989-1992	155	1.5	244	9.3	205	17.2
1999-2002	84	0.8	127	4.4	144	10.6
2007-2010	80	0.7	112	3.3	124	8.0
Uterine cancer						
1969-1972	114	1.2	693	28.4	340	55.1
1979-1982	66	0.7	498	19.7	458	49.9
1989-1992	46	0.5	416	15.8	607	50.9
1999-2002	53	0.5	326	11.2	457	33.6
2007-2010	43	0.4	316	9.3	467	30.1
Ovarian cancer						
1969-1972	321	3.3	823	33.7	304	49.3
1979-1982	281	2.9	892	35.3	496	54.1
1989-1992	224	2.2	816	31.0	718	60.2
1999-2002	165	1.6	713	24.4	717	52.7
2007-2010	165	1.5	790	23.3	775	49.9

*PY* Person Years

Mortality rates continuously decreased for cervical and uterine cancer, and for ovarian cancer in the <55 year olds. For breast cancer and the other age groups of ovarian cancer, mortality rates decreased only as from 1979–1982 and from 1989–1992 for 75+ year olds respectively.

Table 2 shows the results of the spatio-temporal regression analysis by cancer site and age group. With the spatial analysis, we could confirm the time trends observed in the crude rates in Table 1, while only in few cases the covariates had a significant effect on the standardized mortality ratio (SMR). Language region had in none of the models a significant effect on mortality, urbanisation only in 3 models: An urban environment was associated with a significantly lower mortality of 55–74 year olds in uterine cancer and <55 year olds in ovarian cancer, and associated with higher ovarian cancer mortality in 75+ year olds.

**Table 2:** Spatio-temporal model estimates of age specific female cancer mortality in Switzerland from 1969–1972 to 2007-2010

	SMR Ratio (95 % Bayesian Credible Interval)							SMR Ratio (95 % Bayesian Credible Interval)					
Age group	*<55*		*55-74*		*75+*		Age group	*<55*		*55-74*		*75+*	
Breast cancer							Uterine cancer						
Period							Period						
1969-1972	1.00		1.00		1.00		1969-1972	1.00		1.00		1.00	
1979-1982	0.97	(0.89;1.06)	1.03	(0.97;1.09)	1.03	(0.95;1.12)	1979-1982	**0.52**	(0.38;0.71)	**0.68**	(0.61;0.76)	0.89	(0.77;1.03)
1989-1992	**0.90**	(0.83;0.98)	1.00	(0.94;1.06)	**1.22**	(1.14;1.32)	1989-1992	**0.33**	(0.23;0.45)	**0.55**	(0.49;0.63)	0.88	(0.77;1.02)
1999-2002	**0.64**	(0.59;0.70)	**0.84**	(0.80;0.89)	**0.91**	(0.84;0.98)	1999-2002	**0.32**	(0.23;0.45)	**0.39**	(0.35;0.45)	**0.57**	(0.49;0.66)
2007-2010	**0.50**	(0.46;0.55)	**0.77**	(0.73;0.81)	**0.91**	(0.84;0.98)	2007-2010	**0.23**	(0.16;0.33)	**0.33**	(0.29;0.38)	**0.51**	(0.44;0.59)
Language							Language						
German	1.00		1.00		1.00		German	1.00		1.00		1.00	
French	1.09	(0.89;1.32)	0.95	(0.83;1.09)	1.07	(0.92;1.25)	French	1.16	(0.73;1.89)	1.25	(0.99;1.63)	1.00	(0.79;1.29)
Italian/Roman.	0.96	(0.71;1.34)	0.97	(0.77;1.22)	1.01	(0.80;1.29)	Italian/Roman.	1.10	(0.44;2.43)	0.92	(0.57;1.40)	0.93	(0.59;1.44)
Urbanisation level							Urbanisation level						
Rural	1.00		1.00		1.00		Rural	1.00		1.00		1.00	
Urban	1.08	(1.00;1.18)	1.04	(0.99;1.10)	1.01	(0.96;1.07)	Urban	0.99	(0.76;1.33)	**0.89**	(0.81;0.99)	1.00	(0.89;1.11)
Cervical cancer							Ovarian cancer						
Period							Period						
1969-1972	1.00		1.00		1.00		1969-1972	1.00		1.00		1.00	
1979-1982	**0.65**	(0.55;0.77)	**0.80**	(0.70;0.91)	**0.76**	(0.62;0.92)	1979-1982	**0.81**	(0.69;0.95)	1.04	(0.94;1.14)	1.09	(0.94;1.25)
1989-1992	**0.39**	(0.32;0.46)	**0.49**	(0.41;0.58)	**0.55**	(0.45;0.68)	1989-1992	**0.57**	(0.48;0.68)	0.91	(0.83;1.00)	**1.20**	(1.05;1.38)
1999-2002	**0.18**	(0.14;0.23)	**0.23**	(0.19;0.28)	**0.34**	(0.27;0.41)	1999-2002	**0.37**	(0.30;0.44)	**0.73**	(0.66;0.81)	1.06	(0.92;1.21)
2007-2010	**0.15**	(0.12;0.20)	**0.18**	(0.14;0.22)	**0.25**	(0.20;0.31)	2007-2010	**0.32**	(0.26;0.38)	**0.70**	(0.63;0.77)	1.00	(0.88;1.14)
Language							Language						
German	1.00		1.00		1.00		German	1.00		1.00		1.00	
French	0.98	(0.70;1.35)	0.97	(0.69;1.30)	0.95	(0.67;1.37)	French	0.91	(0.68;1.25)	0.98	(0.81;1.18)	0.93	(0.74;1.16)
Italian/Roman.	0.81	(0.41;1.45)	1.08	(0.64;1.75)	1.47	(0.81;2.78)	Italian/Roman.	1.17	(0.64;1.92)	1.00	(0.71;1.39)	0.72	(0.50;1.06)
Urbanisation level							Urbanisation level						
Rural	1.00		1.00		1.00		Rural	1.00		1.00		1.00	
Urban	1.11	(0.94;1.33)	1.07	(0.92;1.24)	1.03	(0.87;1.23)	Urban	**0.85**	(0.74;0.99)	1.04	(0.96;1.13)	**1.13**	(1.02;1.25)
	Spatial variation (95 % Bayesian Credible Interval)							Spatial variation (95 % Bayesian Credible Interval)					
Age group	*<55*		*55-74*		*75+*		Age group	*<55*		*55-74*		*75+*	
Breast cancer	0.27	(0.22;0.33)	0.23	(0.19;0.27)	0.25	(0.21;0.29)	Uterine cancer	0.46	(0.32;0.67)	0.35	(0.28;0.44)	0.33	(0.26;0.43)
Cervical cancer	0.41	(0.32;0.54)	0.36	(0.28;0.47)	0.41	(0.31;0.54)	Ovarian cancer	0.36	(0.27;0.46)	0.29	(0.24;0.36)	0.32	(0.26;0.41)

Results from model 1 (cf. Table 3). Bold values denote Age-Standardized Mortality-Ratio (SMR) Ratios significantly different from 1. Spatial variation (standard deviation of spatial random effects): a value of 0 means that there is no spatial correlation

In the elderly (75+ year olds), a significant increase in breast and ovarian cancer mortality until 1989–1992 was observed and decreasing only since then (Tables 1 and 2).

**Fig. 2:**
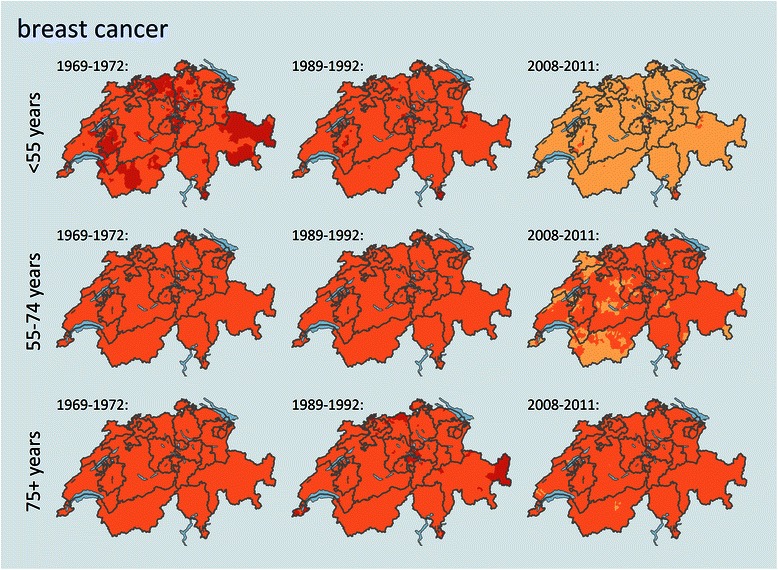
Trends and geographical distribution of age standardized breast cancer mortality (SMR) by age group and among selected time periods. Values are calculated and smoothed in relation to the cancer site and age specific all period combined mortality. Darker colours represent a higher mortality for the specific age structure and population in that area and time period, a detailed color key is provided in additional file 2.

**Fig. 3:**
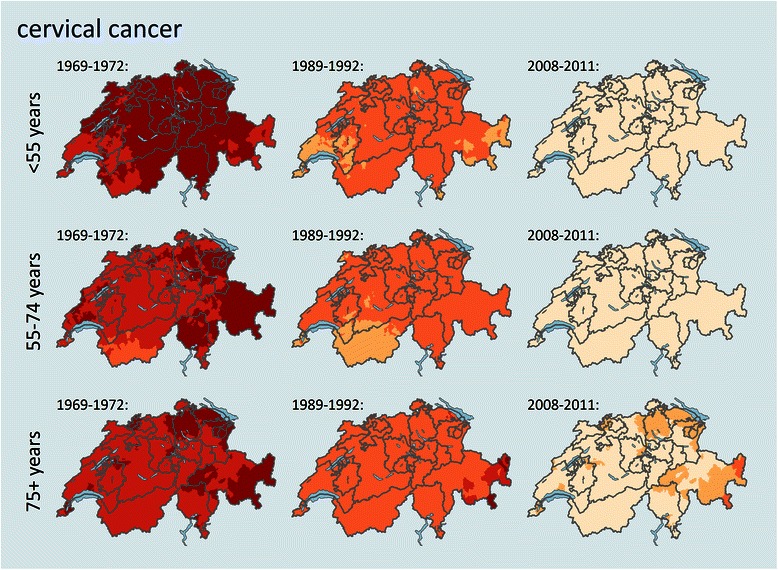
Trends and geographical distribution of age standardized cervical cancer mortality (SMR) by age group and among selected time periods. Values are calculated and smoothed in relation to the cancer site and age specific all period combined mortality. Darker colours represent a higher mortality for the specific age structure and population in that area and time period, a detailed color key is provided in additional file 2.

**Fig. 4:**
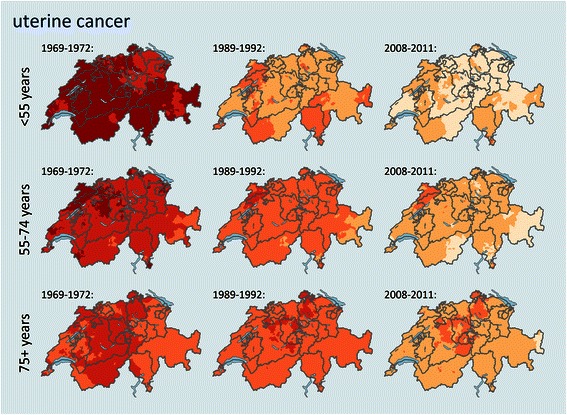
Trends and geographical distribution of age standardized uterine cancer mortality (SMR) by age group and among selected time periods. Values are calculated and smoothed in relation to the cancer site and age specific all period combined mortality. Darker colours represent a higher mortality for the specific age structure and population in that area and time period, a detailed color key is provided in additional file 2.

**Fig. 5:**
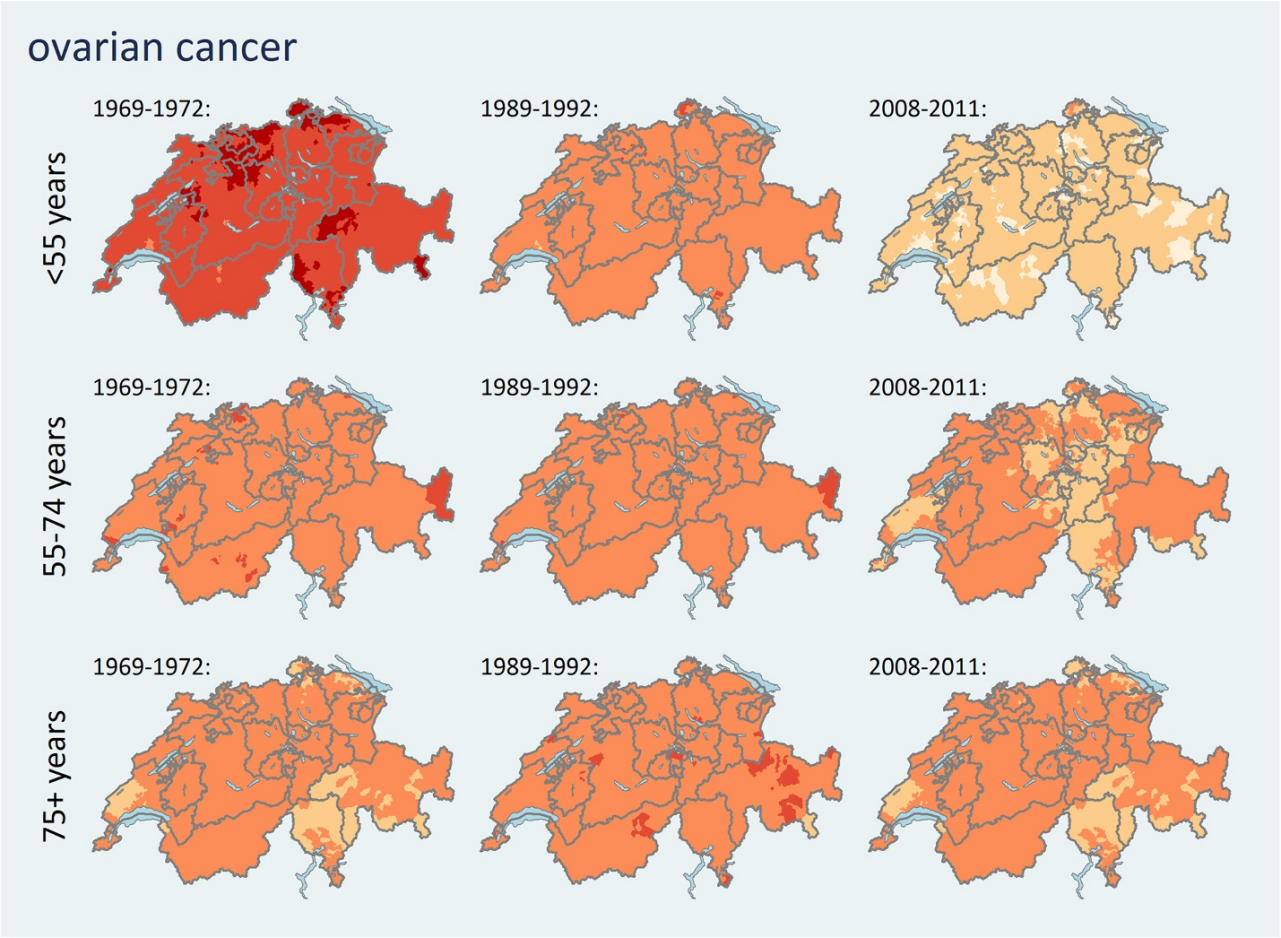
Trends and geographical distribution of age standardized ovarian cancer mortality (SMR) by age group and among selected time periods. Values are calculated and smoothed in relation to the cancer site and age specific all period combined mortality. Darker colours represent a higher mortality for the specific age structure and population in that area and time period, a detailed color key is provided in additional file 2.

The spatial patterns of mortality based on smoothed small area estimates (Figs. 2, 3, 4 and 5, Additional file 1) are different for the female cancers and age groups and not homogenous among the country. No general, significant coincidence with cantonal or language region borders could be observed, with the latter additionally being confirmed by spatial regression for all cancer sites and age groups (Table 2). The spatial patterns form either sub-cantonal areas or canton-overspanning areas.

For all cancer sites and age group combinations the model 1 with Poisson distributed data and only one, spatially structured, random effect was identified as the best model, with lowest DIC (see Table 3). SMR ratios in the non-spatial models were close to the results presented in Table 2, and significance was the same for all but 4 out of 84 coefficients, with their CIs being close to zero in both models.

**Table 3:** Model selection based on Deviance Information Criterion (DIC)

Deviance Information Criterion (DIC)												
Age group	*<55*	*55-74*	*75+*	*<55*	*55-74*	*75+*	*<55*	*55-74*	*75+*	*<55*	*55-74*	*75 N*
Model	Breast cancer			Uterine cancer			Cervical cancer			Ovarian cancer		
**1: P/1re**	**13,430**	**20,328**	**18,161**	**2,140**	**8,521**	**8,327**	**4,300**	**5,459**	**4,167**	**5,417**	**11,887**	**9,404**
**2: NB/1re**	13,462	20,373	18,216	2,149	8,542	8,345	4,312	5,473	4,179	5,430	11,912	9,432
**3: P/2re**	13,457	20,371	18,212	2,142	8,539	8,345	4,309	5,469	4,180	5,431	11,920	9,424
**4: NBN2re**	13,494	20,428	18,275	2,149	8,562	8,371	4,325	5,488	4,196	5,449	11,951	9,457

Lowest DIC values per cancer site and age group are highlighted in bold face. Models 1 and 3 are Poisson regression models (P), models 2 and 4 negative binomial (NB). Models 1 and 2 have one spatially structured random effect (re), models 3 and 4 an additional, unstructured random effect

## Discussion

Using modern Bayesian small area modelling and mapping techniques we have been able to show that all investigated groups of women in Switzerland have benefited from progress in cancer control regardless of place of residence in the past 40 years. We observed only small differences in the geographical variation of mortality.

A factor, which may have contributed to breast and uterine cancer mortality reductions, is the change in the use of hormone replacement therapy (HRT) [7]. After an association of HRT use with breast cancer occurrence was reported [8], its use declined sharply.

We were also not able to show similar spatial patterns in breast and ovarian cancer mortality although they share several life style related, environmental and genetic risk factors. It should be noted however, that hereditary cancer accounts only for about 5-10 % of the cases in breast cancer [9] and about 15 % in ovarian cancer [10]. They are shown to occur at younger age and more advanced stage; still, a visible effect on the mortality map may only be seen in areas with ethnic groups or very large families with a highly elevated risk for hereditary cancer. Such a risk has been described for Ashkenazi Jewish women. The BRCA Ashkenazi founder gene mutations are prevalent in approximately 2 % of these women [11] with communities of Ashkenazi mainly found in urban areas; largest communities are in the cities of Zürich, Geneva and Basel contributing to 1-2 % of the population [12, 13]. However, the breast and ovarian cancer risk in BRCA carriers is affected by genetic modifiers and non-genetic factors, for example, reproductive behaviour, hormonal exposure, lifestyle and risk reduction surgeries [14]. We could not observe an elevated mortality for the three cities in contrast to the surrounding area and it remains unclear to which extent the mortality rates are driven by these hereditary forms of cancer.

Considerable differences in health and health related behaviour have been reported for the Swiss language regions including alcohol intake, smoking and a healthy diet [15, 16] but lacked significance as regression factors in our analysis.

Only for three cancer site-age group combinations was the urbanisation level identified as a significant factor. Urbanisation is serving as a proxy for access to and quality of medical services, education and health consciousness [3]. By our regression with 20 years of new data, we could not formally confirm an urban–rural gradient for breast cancer as described by Schüler & Bopp [3] as significant.

Overall, no general pattern across age groups or cancer sites was present.

The reduction of mortality was stronger in the younger age groups, which is probably the result of better survival and therefore a shift in the age of death. This would also explain the temporary increase in breast and ovarian cancer death risk around the year 1990 in the 75+ year olds. In addition, in this age group multi-morbid conditions and fewer treatments are common [17]. Sant et al. [18] noted that poor survival for gynaecological cancers in the elderly could be due to advanced stage at diagnosis, or failure to give adequate treatment, perhaps because of comorbidity. In general, the interpretability of results in this age group is limited due to its small size, more multi-morbid conditions together with possible inconsistencies in death certification over time, because of only allowing one single cause of death.

### Strengths and limitations

As cancer deaths are rare events and in order to increase the power, different geographical units have been used when analysing cancer mortality data in the past. Some authors have used selected cantons [2] and Schüler & Bopp [3] used for their cancer atlas somewhat smaller mobility regions based on the accessibility to goods and services but which do not take into account population size. As a result, this choice was too aggregated for some urban areas and not aggregated enough for some sparsely populated areas in order to reveal robust, underlying trends. In view that the choice of the geographical unit of analysis may greatly influence results [19], the combination of small geographical units with a state-of-the art smoothing technique enabled a more detailed analysis. With this analysis, we could additionally show the driving age groups or subareas of elevated or reduced mortality in certain regions, while reducing uncertainties due to small numbers and adding an investigation of non-linear time trends.

In general, smoothing allows an estimation of the underlying risk, in a sort of a long-year average, rather than the actual situation. However, for single municipalities, without fully eliminating it, the use of Bayesian smoothing reduces the probability to detect narrow areas with specifically high or low risk. Municipalities at the country border may not benefit from smoothing to the same extent as municipalities in the interior of the country due to unknown data on the other side of the border. Therefore, in the interpretation of the results emphasis should be given to the broader spatial patterns rather than to single municipalities.

Comparing with the previous work of Schüler & Bopp [3] our study not only extended their work by 20 more years and corrected for non-linear time effects, more importantly, we were able to correct the foreseen overestimation in mortality numbers until 1994, which could not be adequately addressed earlier. Priority rules in the coding of causes of death led to an overestimation in cancer deaths due to their prioritization over other comorbidities. The applied methodology of age standardisation takes advantage of the actual age structure rather than a standard population.

There are important limitations to our study. Risk factors affect incidence but are not necessarily linked to mortality [20]. The progression stage of the tumours and their histological type could not be taken into account, as the ICD-classification does not include histological type for the sites studied. The regional case mix and its changes over time therefore may have distorted the results.

Further distortions may arise from the uncertainty as to what level the reported main cause of death and comorbidities are comparable in time and between regions, although the central coding speaks in favour of a certain homogeneity in the coding procedure. In the elderly with frequent multi-morbid conditions, the probability of misclassification is higher.

Furthermore, after prior analysis the covariates language region and urbanisation level were fixed in time for the municipalities, so that varying developments therein may have resulted in inaccuracies.

## Conclusions

Female gender related cancer mortality continuously decreased in Switzerland. In most age groups, this decline was significant and quite strong in the past decades, resulting in values more than 6 times lower within 40 years. The strongest reduction of mortality was observed for cervical cancer, followed by uterine, ovarian and breast cancer.

Geographical differences are small and do not follow cantonal borders. Spatial patterns were different for each cancer site and age group. The reasons for these differences are manifold, rising awareness, major advances in cancer therapy and ongoing developments in the field had a major impact on the cancer mortality.

Information on the geographical patterns and temporal trends of the disease burden at different regional scales are important for the design, implementation and evaluation of programs for cancer control. Access to specialized medical facilities should be increased especially in high priority areas in order to further reduce disparities. However, existing disparities are small.

## Appendix

### Appendix model formulations

Observed age and cancer site-specific counts of deaths *Y*_*it*_ in municipality *i*(*i* = 1, …, *N*) in period *t* to follow a poisson distribution *Y*_*it*_ ~ *Pois*(*μ*_*it*_). Age and cancer specific random effects as well as possible non-linear trends were modelled on the log of the mean Age Standardized Mortality Ratio (SMR).

$$ \log \left({\mu}_{it}\right)= \log \left({E}_{it}\right)+\alpha +{X}_{ij}^T{\beta}_s+{\Phi}_i $$

where *E*_*it*_ is the age and cancer specific expected number of deaths, *X*_*is*_ the vector of covariates *s* related to municipality *i* and *β*_s_ the coefficients of associated covariates. Time periods are included as covariates. Spatial correlation by age and cancer specific random effects Φ_*i*_ on municipality level *i*, modelled via a Conditional Autoregressive (CAR) process. Spatial dependency among the municipalities was introduced by the conditional prior distribution of Φ_*i*_ with

$$ {\Phi}_i\sim N\left(\frac{\gamma {\displaystyle {\sum}_{\begin{array}{c}\hfill q=1\hfill \\ {}\hfill q\ne i\hfill \end{array}}^N}{c}_{iq}{\Phi}_q}{w_i},,,\frac{\sigma^2}{w_i}\right) $$

where *c*_*iq*_ characterizes the degree of spatial influence of municipality *i* to the remaining municipalities, *γ* quantifying the overall spatial dependence and *w*_*i*_ being the number of neighbours of municipality *i*. We used the intrinsic version of this CAR model as proposed by Besag, York and Mollie (1991) where *c*_*iq*_ takes the value 1 if municipalities are adjacent and 0 otherwise, and *γ* being equal to one. As further prior distributions we used:

$$ \frac{1}{\sigma^2}\sim \varGamma \left(2.01,\ 1.01\right), \kern0.5em \alpha \sim U\left(-\infty, +\infty \right), \kern0.5em {\beta}_s\sim N\left(0,\ 0.01\right) $$

## Additional files

Additional file 1: **Detailed Figures of SMR development by cancer sites and age groups.** Development of age standardized breast (Figures S2a-c), cervical (Figures S3a-c), uterine (Figures S4a-c) and ovarian (Figures S5a-c) cancer mortality (SMR) and spatial differences therein among all time periods by age group. (PDF 5957 kb)
Additional file 2: **Color key for figures 2-5.** (PDF 164 kb)
