# Supplementary material for: 40 years of progress in female cancer death risk: a Bayesian spatio-temporal mapping analysis in Switzerland
Source: BMC Cancer. 2015 Oct 9;15:666. doi: 10.1186/s12885-015-1660-8 (PMC4600311; doi:10.1186/s12885-015-1660-8)
Supplement: Additional file 1: — Detailed Figures of SMR development by cancer sites and age groups. Development of age standardized breast (Figures S2a-c), cervical (Figures S3a-c), uterine (Figures S4a-c) and ovarian (Figures S5a-c) cancer mortality (SMR) and spatial differences therein among all time periods by age group. (PDF 5957 kb) [file 12885_2015_1660_MOESM1_ESM.pdf]

Additional file 1: Detailed Figures of SMR development by sites and age groups

Figures 2a-5c: *Development of age standardized breast (figures 2a-c), cervical (figures 3a-c), uterine (figures 4a-c) and ovarian (figures 5a-c) cancer mortality (SMR) and spatial differences therein among time by age group. Values are calculated and smoothed in relation to the all period combined mortality. Darker colours represent a higher mortality for the specific age structure and population in that area and time period.*

Smoothed SMR

≤0.5
0.5-0.75
0.75-1.33
1.33-2
>2

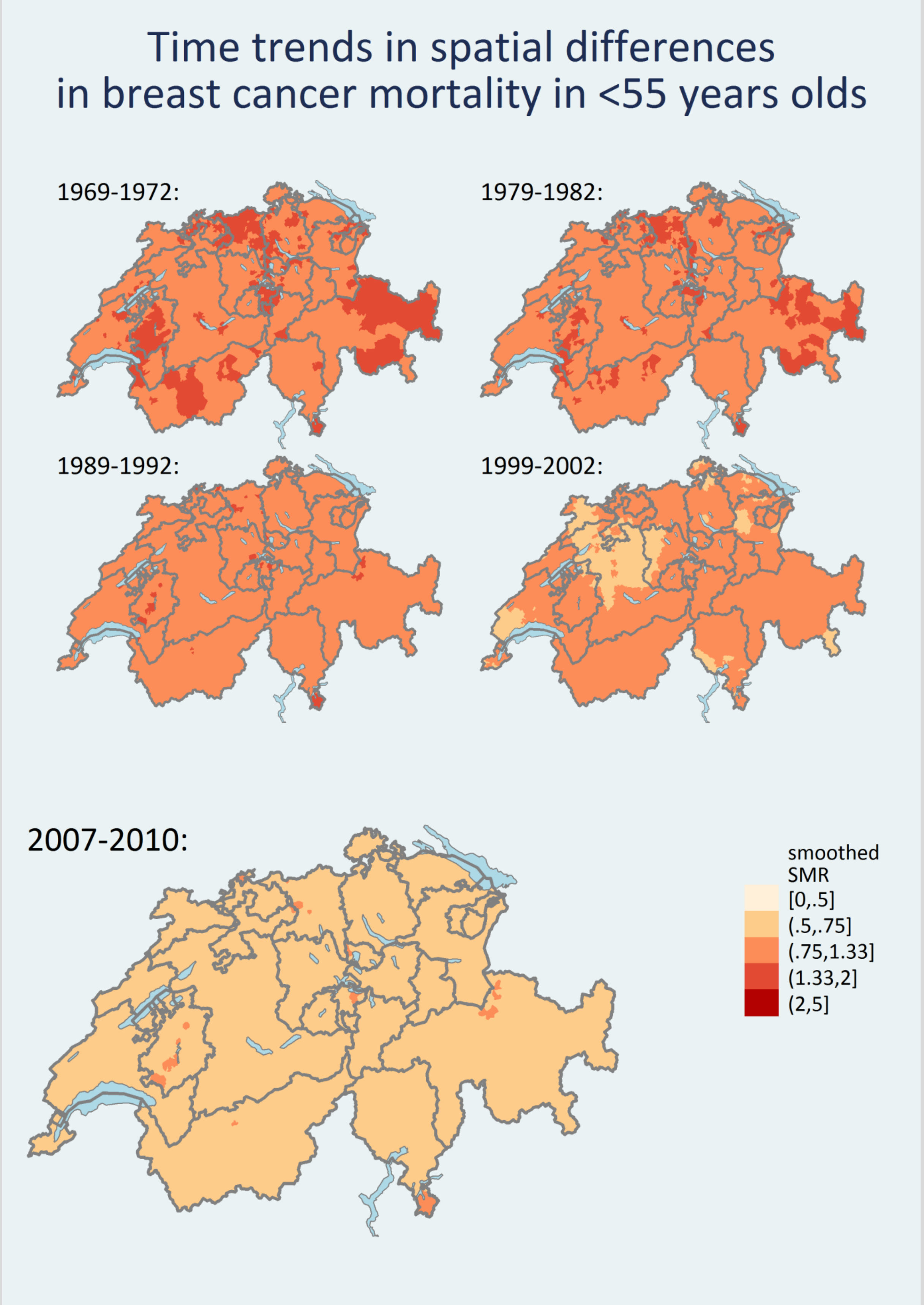

Figure 2a: *Breast cancer, <55 year olds.*

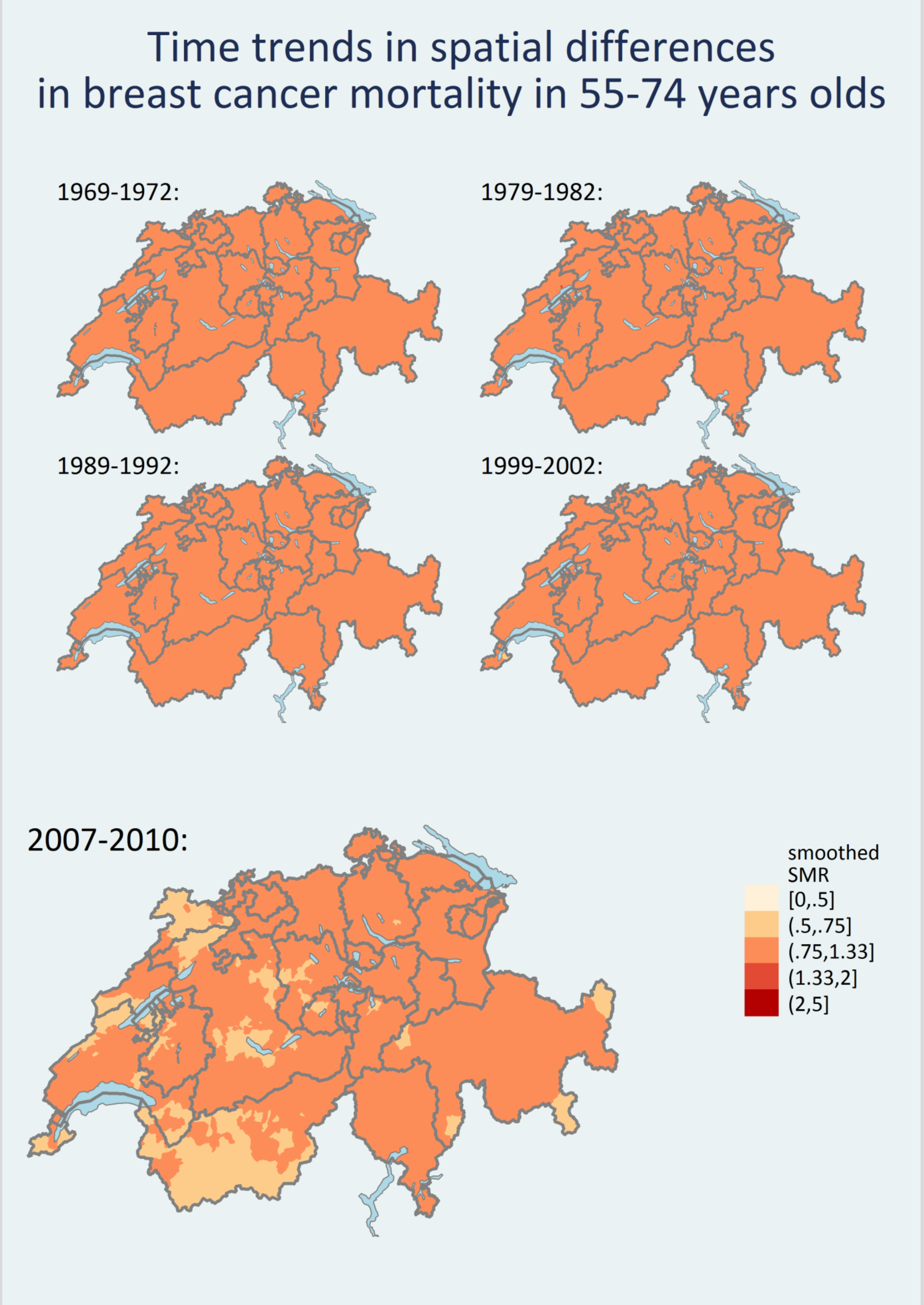

Figure 2b: *Breast cancer, 55-74 year olds.*

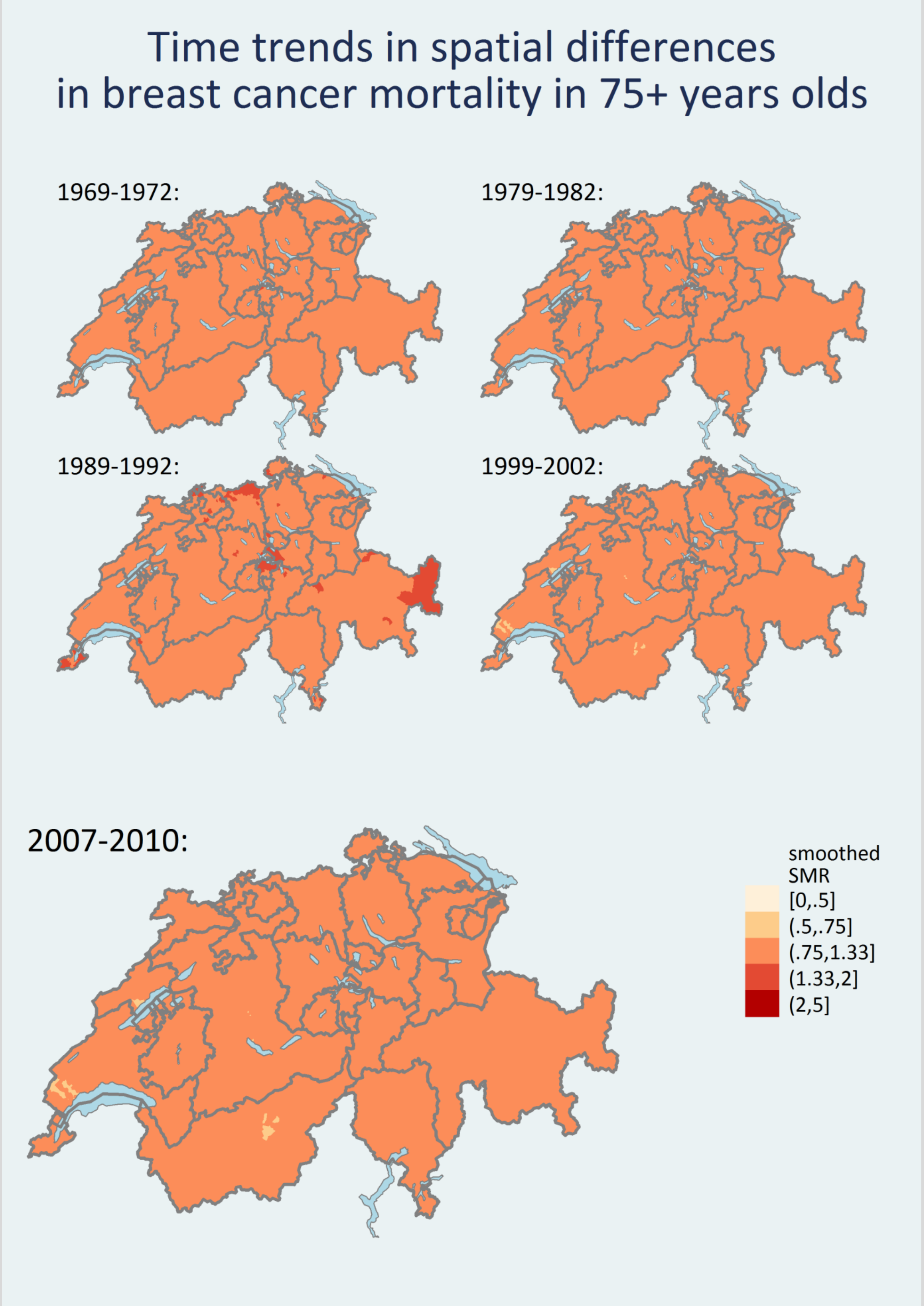

Figure 2c: *Breast cancer, 75+ year olds.*

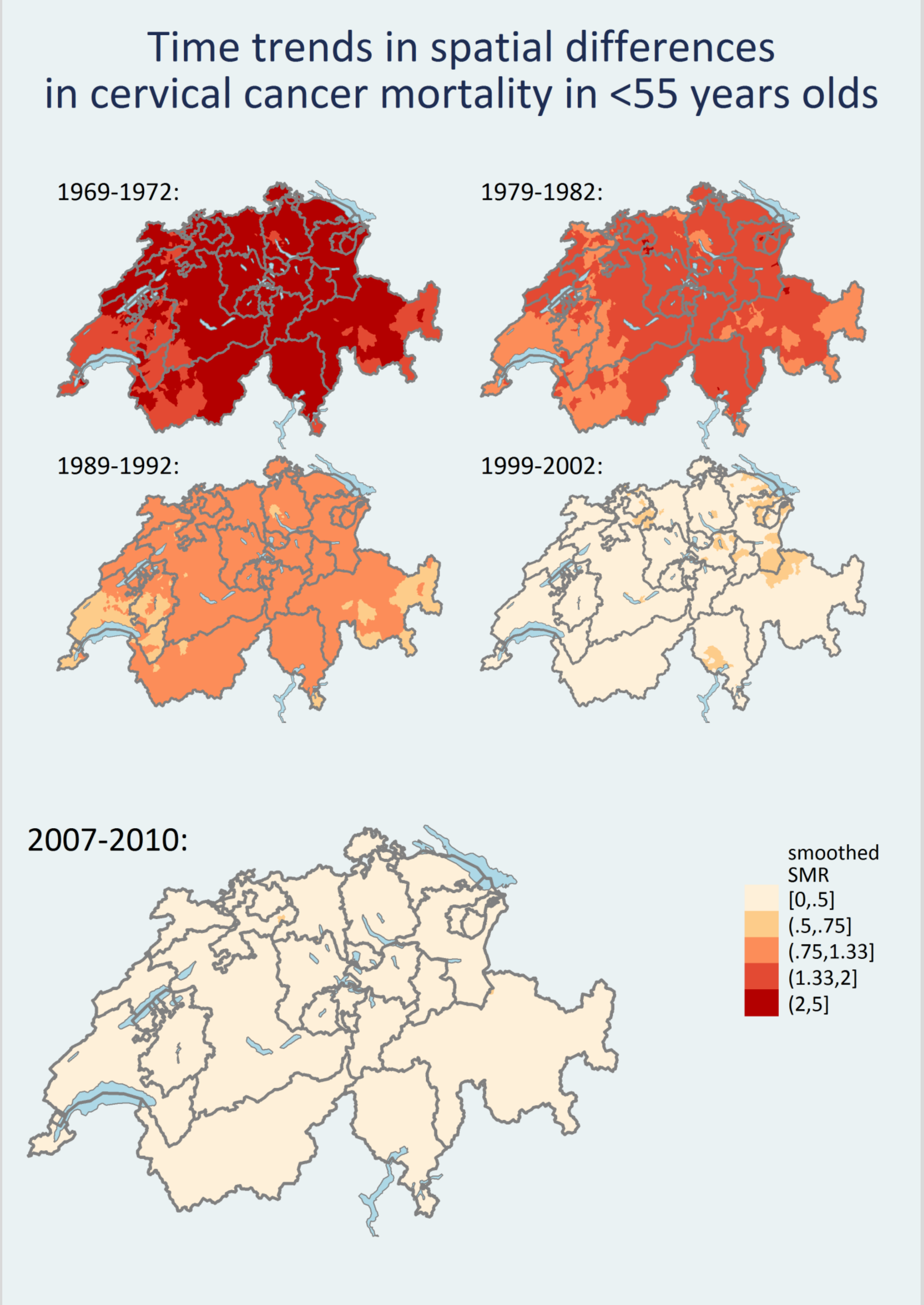

Figure 3a: *Cervical cancer, <55 year olds.*

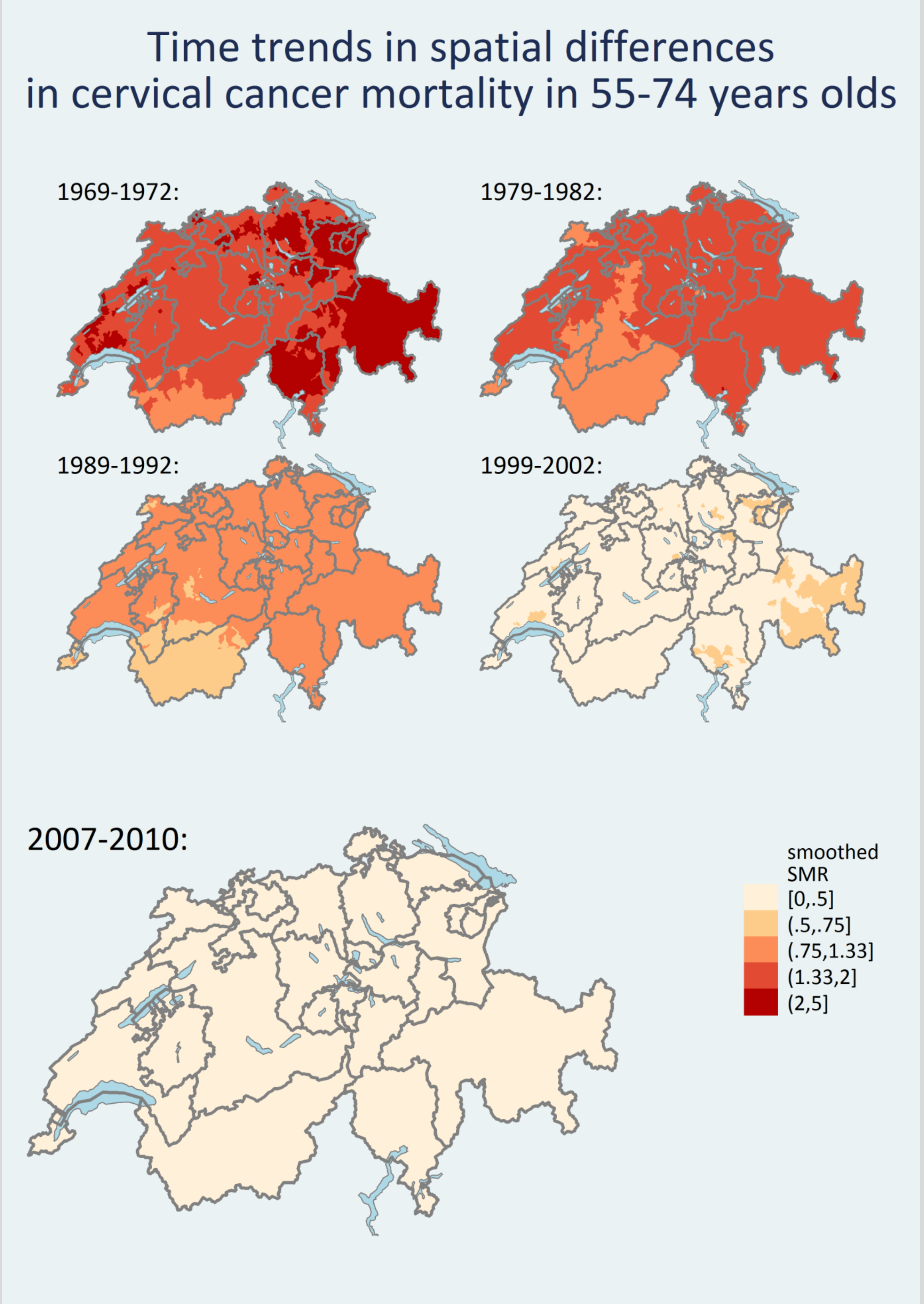

Figure 3b: *Cervical cancer, 55-74 year olds.*

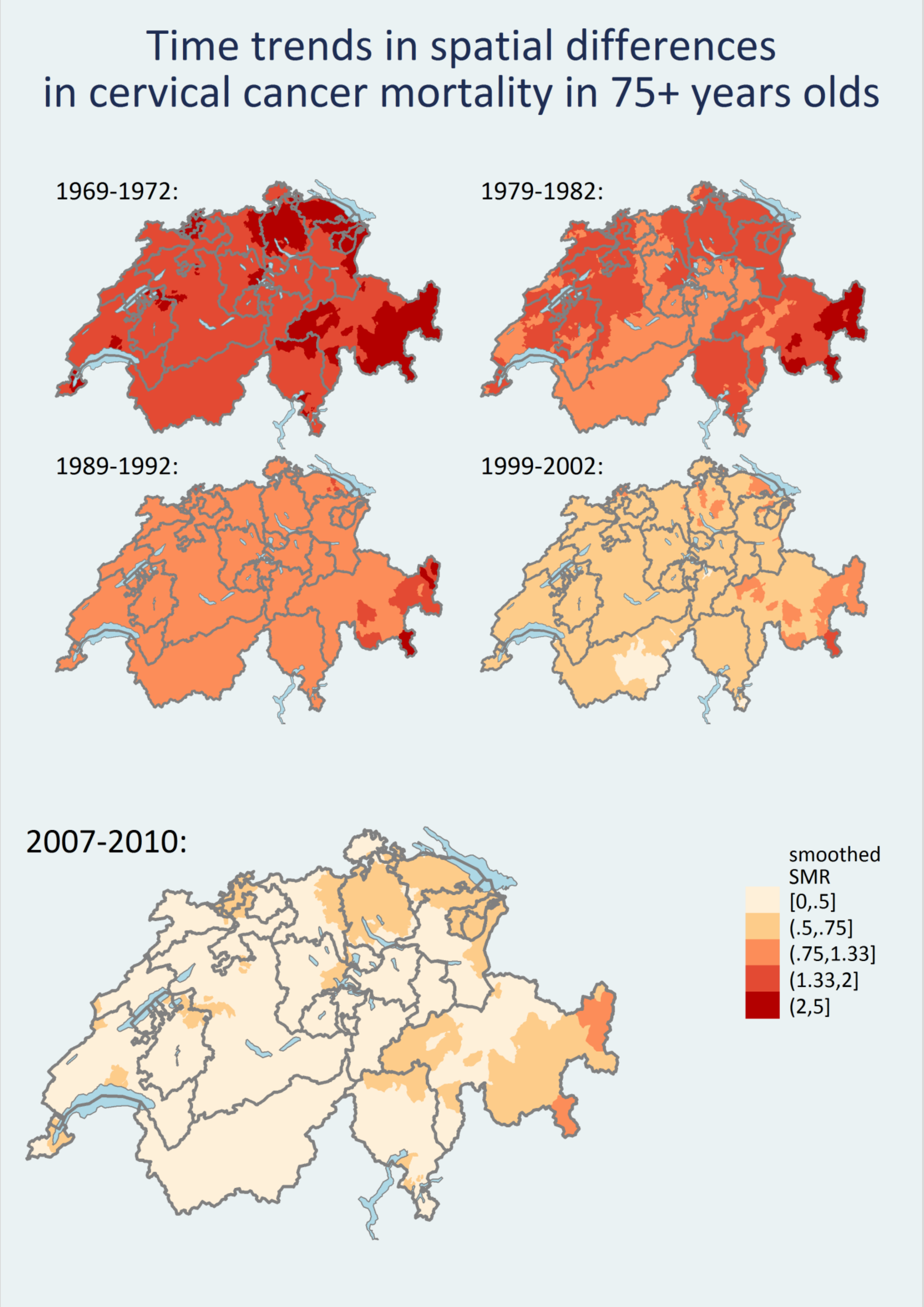

Figure 3c: *Cervical cancer, 75+ year olds.*

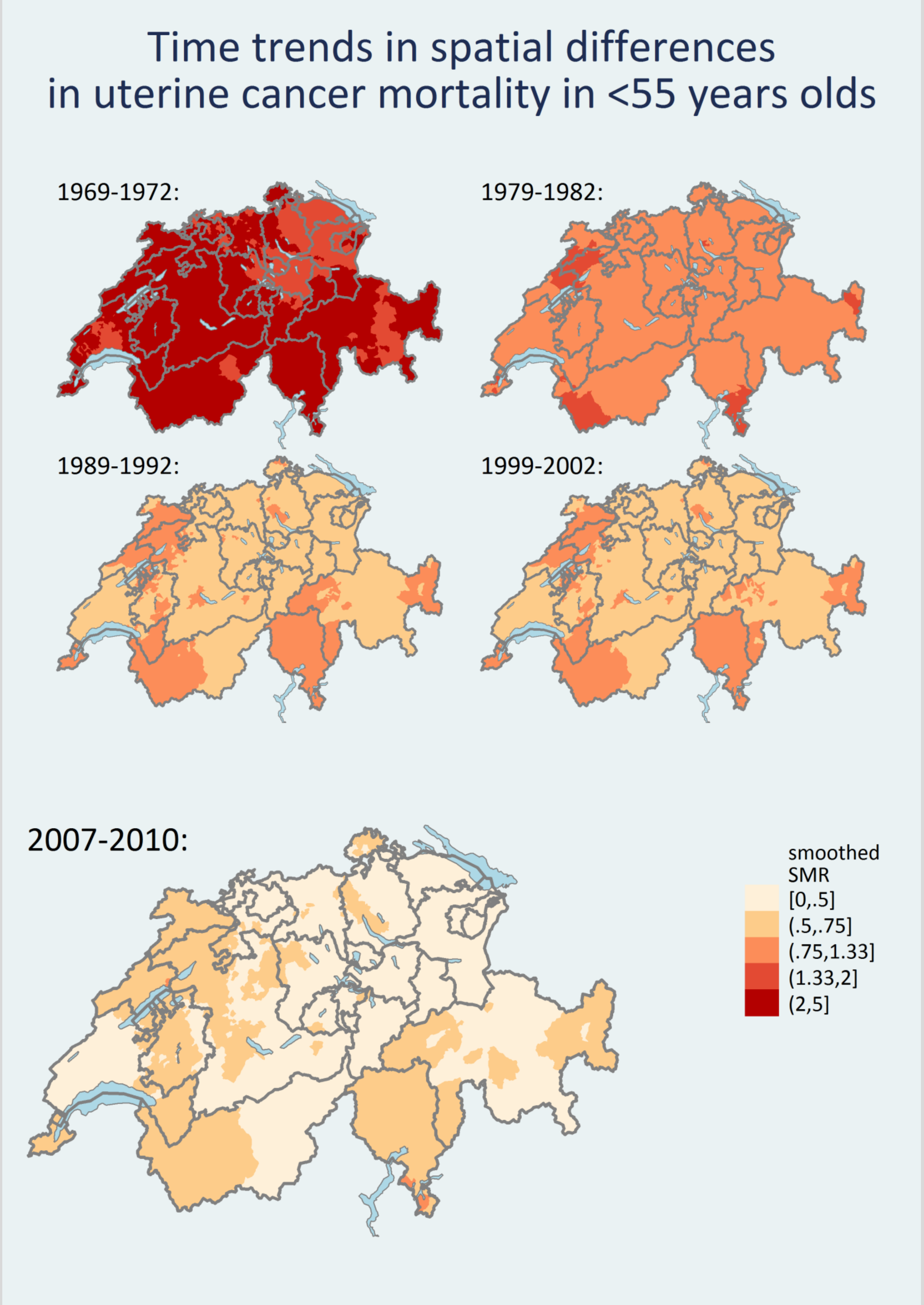

Figure 4a: *Uterine cancer, <55 year olds.*

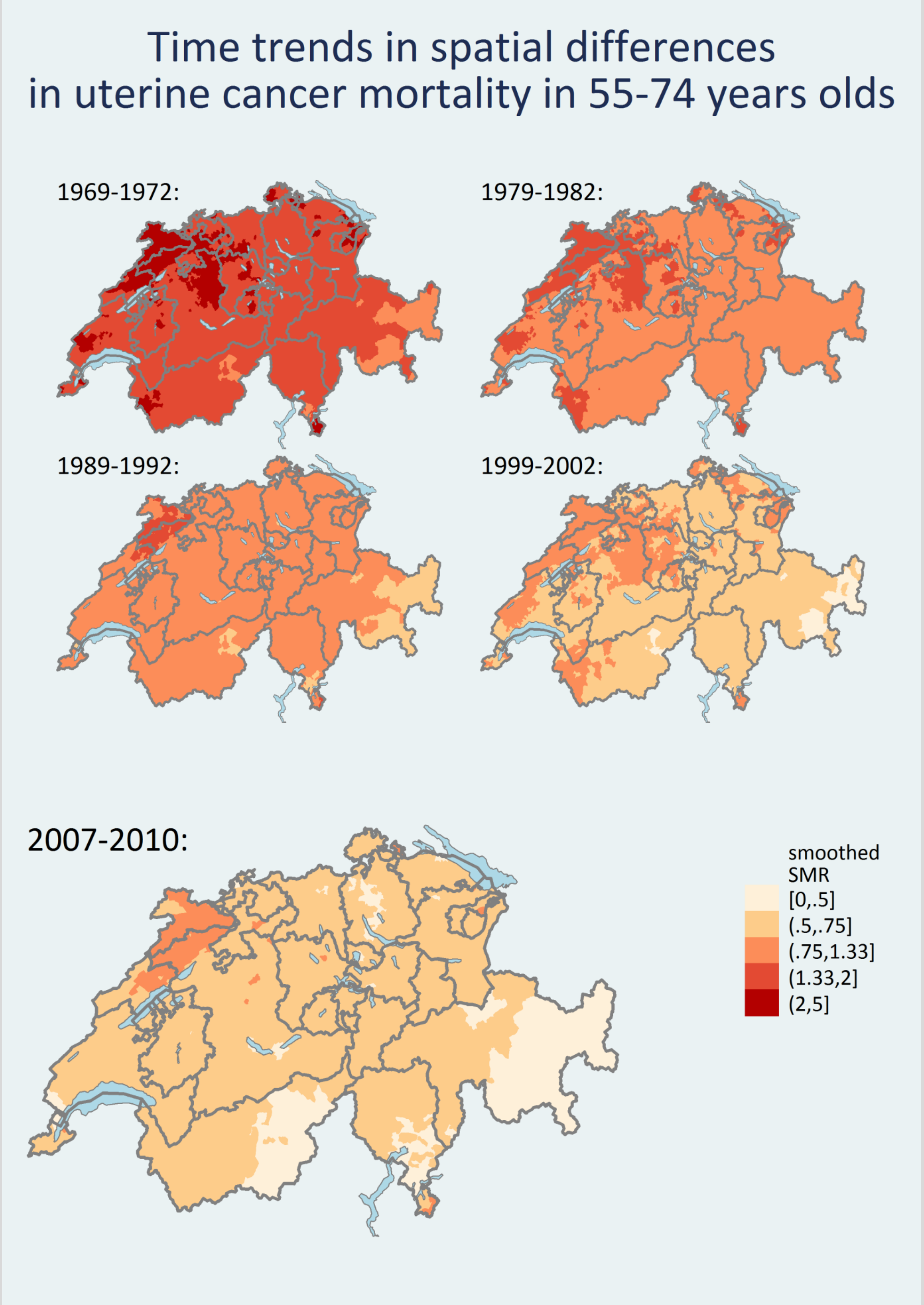

Figure 4b: *Uterine cancer, 55-74 year olds.*

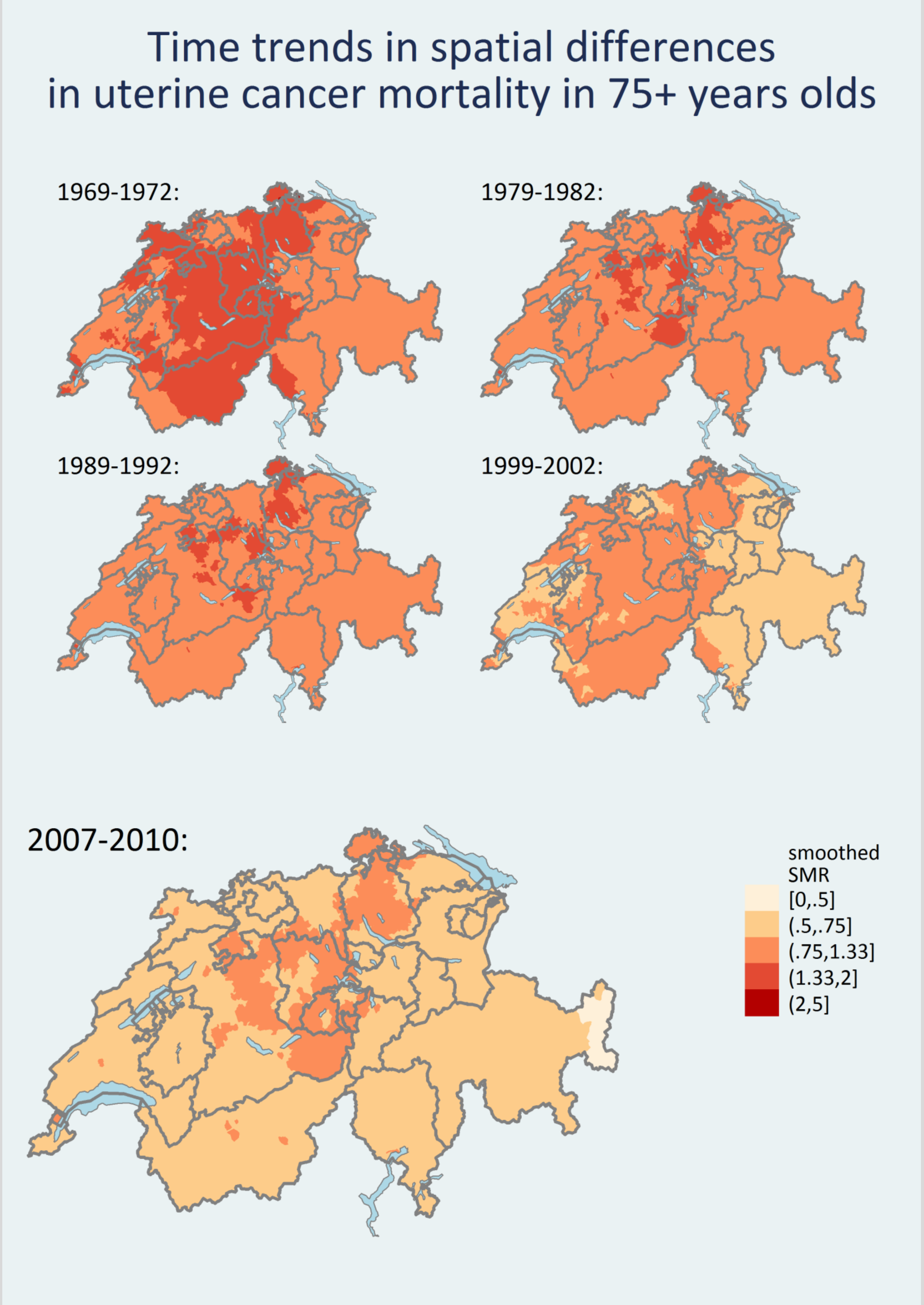

Figure 4c: *Uterine cancer, 75+ year olds.*

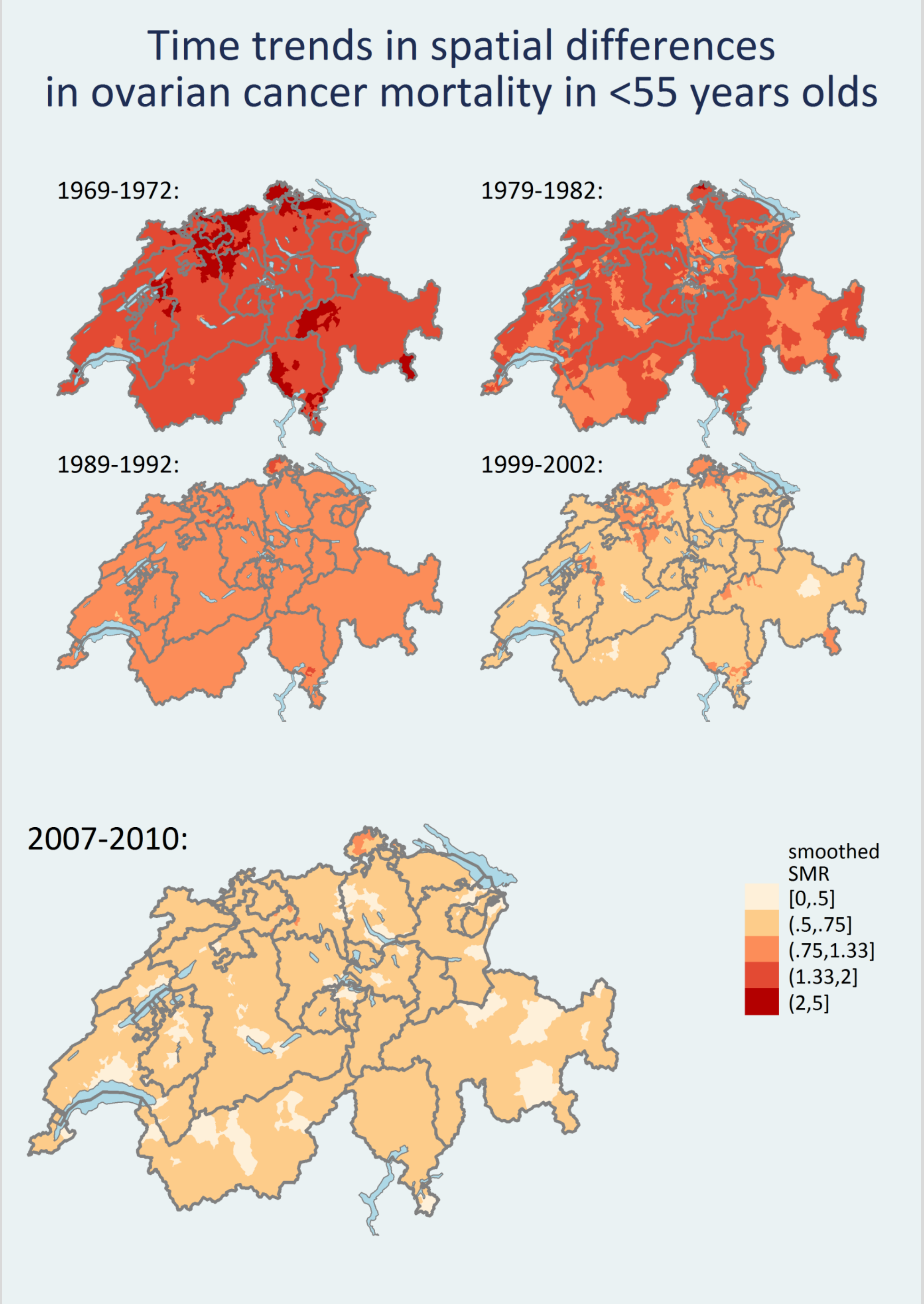

Figure 5a: *Ovarian cancer, <55 year olds.*

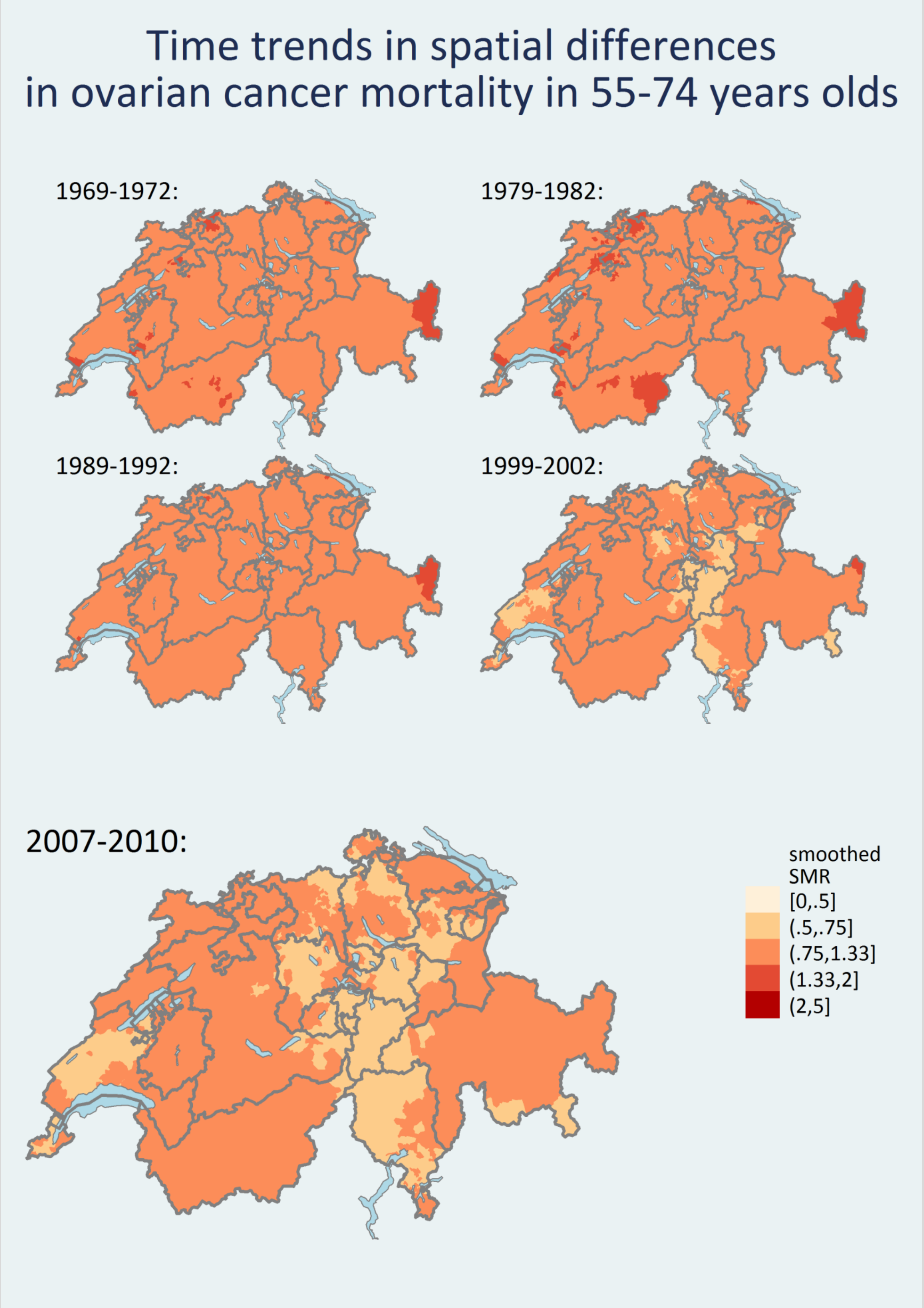

Figure 5b: *Ovarian cancer, 55-74 year olds.*

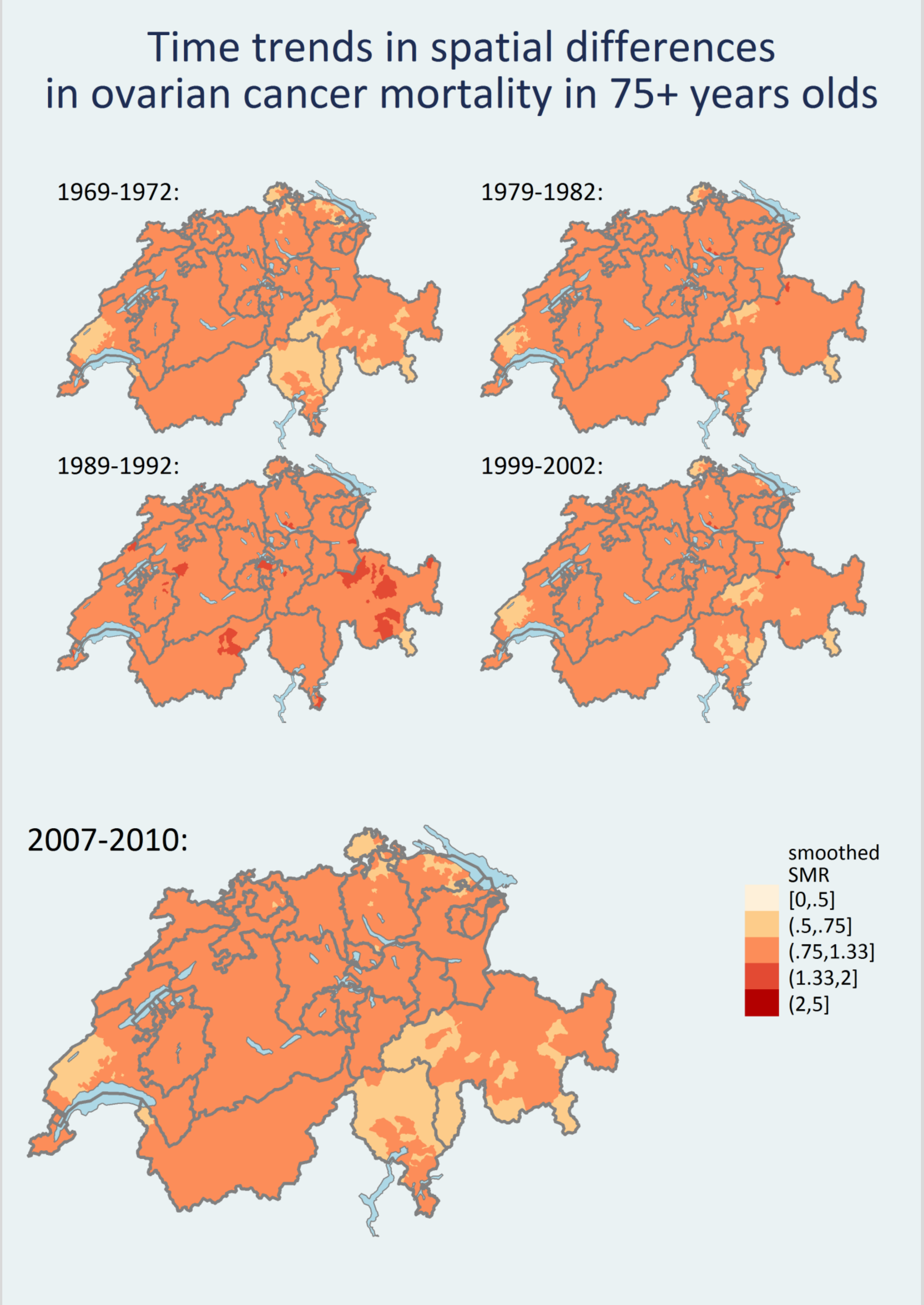

Figure 5c: *Ovarian cancer, 75+ year olds.*
