# Supplementary figures and images for: 40 years of progress in female cancer death risk: a Bayesian spatio-temporal mapping analysis in Switzerland
Source: BMC Cancer. 2015 Oct 9;15:666. doi: 10.1186/s12885-015-1660-8 (PMC4600311; doi:10.1186/s12885-015-1660-8)

# Smoothed SMR

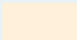

$\leq 0.5$

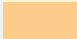

0.5-0.75

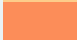

0.75-1.33

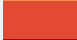

1.33-2

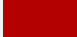

$> 2$

Supplement: Additional file 2: — Color key for figures 2-5. (PDF 164 kb) [file 12885_2015_1660_MOESM2_ESM.pdf]
